# Influence of the Cortical Layer Thickness and Trabecular Layer Pattern Density on 3D-Printed Femur Strength

**DOI:** 10.3390/ma18102187

**Published:** 2025-05-09

**Authors:** Aleksander Znaczko, Krzysztof Żerdzicki, Paweł Kłosowski

**Affiliations:** Department of Structural Mechanics, Faculty of Civil and Environmental Engineering, Gdansk University of Technology, 80-233 Gdansk, Poland; krzysztof.zerdzicki@pg.edu.pl (K.Ż.); klosow@pg.edu.pl (P.K.)

**Keywords:** 3D printing, experimental research, femur, composite, Zortrax M300 Plus, 3D printing patterns, LPD method, Young’s modulus, validation

## Abstract

This paper presents the process of preparing and conducting a uniaxial compression test, developing the results, and determining the compressive strength of a femur made using 3D printing technology. The study considers the variable thickness of the outer layer—imitating cortical bone tissue—and the varying density of the inner layer—imitating trabecular bone tissue—which, with further analysis, may aim to replicate different states of osteoporosis. The compressive strength of the bones varied depending on the thickness of the outer layer and the filling degree. Failure patterns were observed, corresponding to different variants of the produced bones. The predominant failure pattern was the fracture of the femoral head or neck at the proximal end of the femur. The results were compared with previous studies on commercial femur bones, as well as those created using 3D printing technology by other authors. The highest compressive strength was found in the bone with an outer layer thickness of 3.0 mm and 30% infill, with a value of 4778 N. A very similar compressive strength was recorded for the bone with an outer thickness of 2.1 mm and 30% infill, reaching 4519 N. The lowest compressive strength, 2116 N, was observed in the bone with an outer thickness of 1.2 mm and 20% infill.

## 1. Introduction

Three-dimensional (3D) printing technology, also known as additive manufacturing (AM) [1], has found broad applications in various fields of industry and science [2]. These include, among others, the food [3], automotive, and aerospace industries [4]. Three-dimensional printing technology has undergone a multi-stage evolution. The first phase involved its use by architects, artists, and designers who utilized it for creating prototypes or models, which still constitute a significant percentage of its applications. Over time, it has become a key tool in the production of custom components. A characteristic feature of 3D printing is the ease of product replication, design flexibility, and low costs. The future of 3D printing may rely on custom-made products manufactured on demand. Examples of such products include dental and medical devices, as well as spare parts with limited availability. In medicine, the applications of 3D printing include the fabrication of prosthetics, orthotics, implants, surgical models, and tools tailored to the individual needs of patients [5,6,7,8,9,10,11,12,13,14,15,16]. This technology enables the personalization of treatment by matching the shape and biomechanical properties of the produced elements.

In medicine, the applications of 3D printing include the fabrication of prosthetics, orthotics, implants, surgical models, and tools tailored to the individual needs of patients. This technology enables the personalization of treatment by matching the shape and biomechanical properties of the produced elements. In orthopedics, additive manufacturing technology is used for creating custom orthopedic insoles, foot and ankle orthoses, and prosthetic sockets [5,6,7,8,11]. Due to its complex anatomy, 3D printing is also applied in cranio-maxillofacial surgery [9,11]. It is utilized for training purposes and for creating implants that are later filled with non-printable material. Authors L. E. Murr et al. [12] explore the potential for producing patient-specific biomedical implants that replace hard tissues (bones), particularly knee and hip stems, as well as large bone (femoral) intramedullary rods, using AM technology. Furthermore, this technology is also employed for the education and training of doctors and orthopedic specialists [11,13,14].

The wide range of materials used in 3D printing technology includes metals, polymers, composites, ceramics, concrete, and biomaterials [15]. The choice of material depends on application requirements such as mechanical strength, biocompatibility, thermal conductivity, or even water solubility. Metal printing technology is mainly used for research, prototyping, or advanced applications in the aerospace industry [17]. It is also applied in the biomedical and automotive industries [18]. Polymers are the most commonly used materials in the 3D printing industry due to their diversity and ease of adaptation to printing processes. They come in the form of thermoplastic filaments, reactive monomers, resins, or powders. Additive manufacturing (AM) is a crucial method for producing advanced ceramics in tissue engineering and biomaterials. Ceramics are used, for example, to produce scaffolds for bones and teeth [19]. AM technology has also found applications in the construction industry. Contour crafting has been developed as an additive manufacturing method for building structures [20]. It uses significantly larger nozzles and high pressure to extrude concrete paste.

The concept of bio-fabrication includes the production of tissues and organs through bioprinting, bioassembly, and maturation [9,21]. The difference between bio-fabrication and additive manufacturing (AM) lies in the incorporation of cells into the produced biomaterials to create so-called bio-inks [22]. Biomaterials combined with biomolecules and cells mature into the desired shape and form of tissue. They serve as scaffolds and provide signals to shape the tissue structure while biomolecules guide the regeneration process. The use of such materials reduces the risk of rejection of transplanted tissues or organs. Many articles can be found on in vitro or in vivo testing of manufactured cartilage [23], bone [24], aortic valves [25], blood vessels [26], and bioresorbable tracheal splints [27].

Three-dimensional printing technology is used to create models of various human bones. Burkhard M. et al. [28] produced a vertebra model with varying bone density for surgical training. The authors adjusted the thickness of the cortical and cancellous bone in the model, designing five different types of vertebrae. The study displayed haptically and biomechanically realistic simulations of posterior spinal procedures, indicating that these custom-designed vertebrae enable surgical training on spine replicas tailored to specific patients. Clifton W. et al. [29] conducted a study to assess the feasibility of combining 3D-printed models with polymer foam to replicate both the cortical-cancellous vertebral interface and surface anatomy for educational purposes. The study demonstrated that combining 3D-printed vertebral models made of ABS material with porous foam is a viable and effective method for simulating the cortical-cancellous interface of the human vertebral bone in surgical education. Clifton W. et al. [30] investigated the capability of 3D-printed thoracic vertebrae, made from combined thermoplastic polymers, to accurately demonstrate pedicle screw cannulation via the superior articular surface. The study concluded that using 3D-printed thoracic vertebrae combined with thermoplastic polymers is justified. These models can accurately display the anatomical ultrastructure and spatial relationships of elements for safely placing a pedicle screw in the thoracic section. The authors also highlighted the usefulness of this prototyping method in surgical education. F. Metzner et al. [31] developed human femur bone models with a core printed using additive manufacturing (AM) technology. The printed core was coated with a glass fiber composite. The cores were produced in various variants, differing in material and infill density. Compression and three-point bending tests were conducted on the samples, showing good agreement between the mechanical behavior of the printed specimens and human bone. Nägl et al. [32] applied additive manufacturing to produce artificial femur bones. The models were printed using a 3D printer, and their density was mechanically calibrated with different cortical layer thickness variants. The printed models underwent compression tests on the proximal femoral head, and biomechanical behavior was evaluated regarding ultimate strength, stiffness, and fracture patterns. R. Ramesh Kumar et al. [33] used 3D printing technology to produce human femur bone models in three different internal infill patterns: triangular, hexagonal, and line. They also applied three levels of internal layer infill density: 15%, 45%, and 75%. The samples were subjected to tensile testing, thereby determining the maximum tensile force for their specimens.

This study focuses on the analysis of a cantilever structure (column-cantilever structure), using the femur bone as an example. The analyzed structure is a two-layer composite. In each test, the outer layer has a constant thickness, ranging from 1.2 mm to 3.0 mm, depending on the sample, intended to replicate the cortical bone tissue of the human femur. The inner layer, imitating trabecular bone tissue, exhibits variable densities ranging from 10% to 40%. It should be emphasized that the outer layer thickness and the degree of internal densification differ between individual sample variants but remain constant within each variant. The assumption of a constant outer layer thickness represents a simplification compared to real cortical bone, which displays variable thickness both around the cross-section and along the longitudinal axis. Similarly, each sample assumes a uniform density and a consistent internal infill pattern, whereas in reality, the density and structure of trabecular bone tissue exhibit significant variability [34]. This simplification also applies to the experimental setup, which was intentionally simplified due to the innovative nature of this research approach. Future studies are planned to more accurately reflect bone geometry, for example, by introducing variable cortical thickness within a single sample or by employing more complex loading configurations that account, for instance, for muscle forces.

The aim of this work is to evaluate how changes in the thickness of the outer layer, mimicking cortical bone tissue, and the density of the inner layer, mimicking trabecular bone tissue, affect the overall load-bearing capacity and failure patterns of the analyzed structure.

## 2. Materials and Methods

### 2.1. Three-Dimensional Printer Zortrax M300 Plus

**Figure 1:**
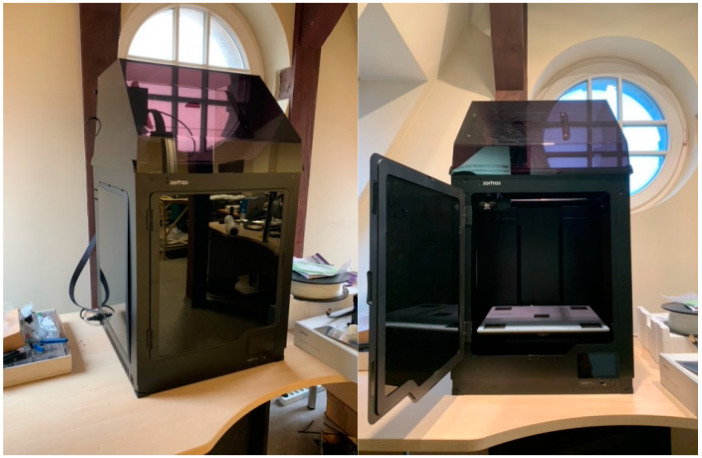
Three-dimensional printer Zortrax M300 Plus.

The 3D printer used for printing bone models is the Zortrax M300 Plus (Zortrax S.A., Olsztyn, Poland) (Figure 1). This printer utilizes additive manufacturing (AM) technology, specifically FFF (Fused Filament Fabrication) or FDM (Fused Deposition Modeling). This technology has previously been used for research on similar models [29,30,31]. The 3D printer was provided for the duration of the research by the Department of Structural Engineering at the Faculty of Civil and Environmental Engineering, Gdańsk University of Technology (Gdańsk, Poland).

### 2.2. Filament Used for Printing

The material used for printing samples for material parameter identification, as well as bone models, is EcoLine PLA (Table 1) by Print-Me (PrintMe, Poland). Based on the literature [29,30,31,32,33], it was selected as the material with properties closest to those of real human bone. EcoLine PLA is made from a biodegradable plastic, polylactide. An advantage of this material is its excellent layer adhesion, allowing for the printing of very large objects. Additionally, this material has no shrinkage, making it suitable for devices without a heated bed.

### 2.3. Three-Dimensional Model Preparation

**Figure 2:**
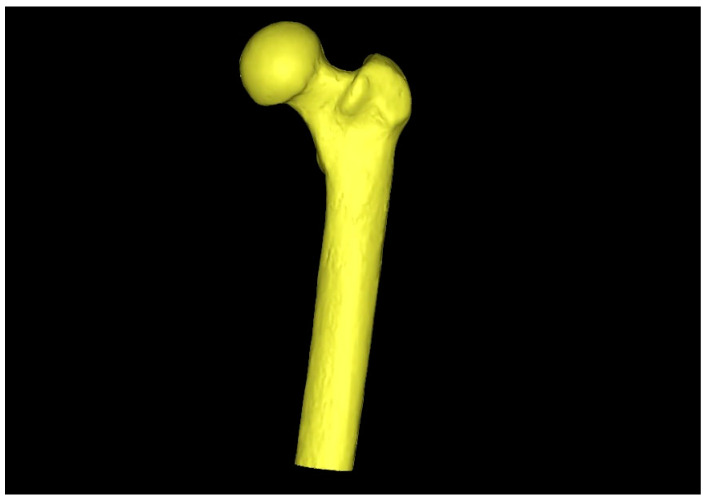
View of the model ready for printing.

The femur bone model by Sawbones (A Pacific Research Company, Sawbones, Pasadena, CA, USA) was generated using CT scan images, which is a common practice in the literature for creating a reliable 3D model [35]. The CT scanner used was the Optima CT660 (GE Healthcare, Chicago, IL, USA). The specialized software MIMICS Materialise 27.0 was used to process the CT scans. With its help, the obtained model was segmented, a 3D reconstruction was performed (Figure 2), and the transformation to an *.STL file was completed.

Using the dedicated Z-Suite 3.6.1 software, models were prepared with varying thicknesses of the outer layer, the cortical layer, and different densities of the inner layer, the cancellous tissue. Models of bones were printed with outer layer thicknesses of 1.2 mm, 2.1 mm, and 3.0 mm. For each mentioned outer layer thickness, a bone was printed with an internal filling imitating cancellous tissue at densities of 10%, 20%, 30%, and 40%.

### 2.4. Laboratory Testing of the Composite Femur Bone Model

**Figure 3:**
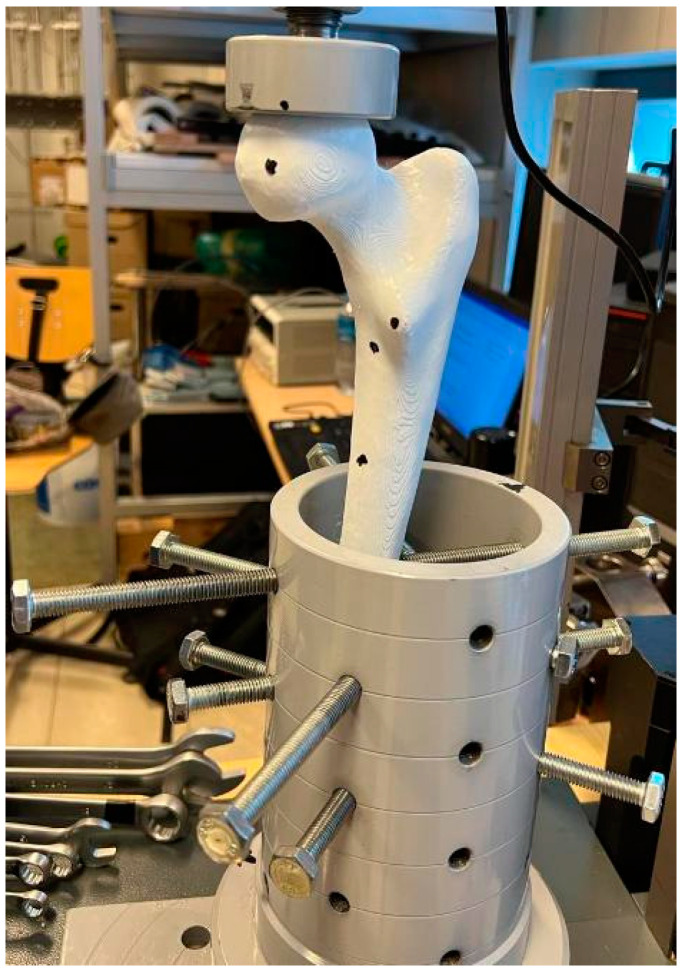
View of the bone.

The uniaxial cyclic compression to failure test for the bone was conducted on a Zwick/Roell Z020 testing machine (Zwick/Roell, Ulm, Germany). The lower part of the bone was inserted into a cylinder and clamped with screws (Figure 3). The load was applied using a dedicated fixture for this test, which has a recess for the femoral head. Both the femoral head at the load application point and the piston were lubricated to minimize friction. The initial force (to eliminate rigid movements inside the system) was set at 100 N, with the application speed of the initial force set to 1 mm/min, and the same value was used for the application speed of the target force. The test was conducted until the complete failure of the sample, i.e., its fracture.

The cyclic axial force applied to the bone included a total of 25 cycles, with the final 25th cycle conducted until the bone’s failure. As mentioned above, the test started with the application of a 100 N force, which served as the initial force for each cycle. The loading was then applied in three phases: Phase 1–10 cycles aimed at stabilizing the bone, with force ranging from 100 N to 500 N. Phase 2–14 cycles, in which the force was increased by 100 N in each subsequent cycle. In this phase, the forces ranged from 600 N to 1900 N. Phase 3—the 25th cycle, in which the maximum force was applied until the bone was destroyed. In the case of variants with an inner layer fill level of 10% and an outer thickness of 1.2 mm and 2.1 mm, the samples failed already in Phase 2.

## 3. Results

**Figure 4:**
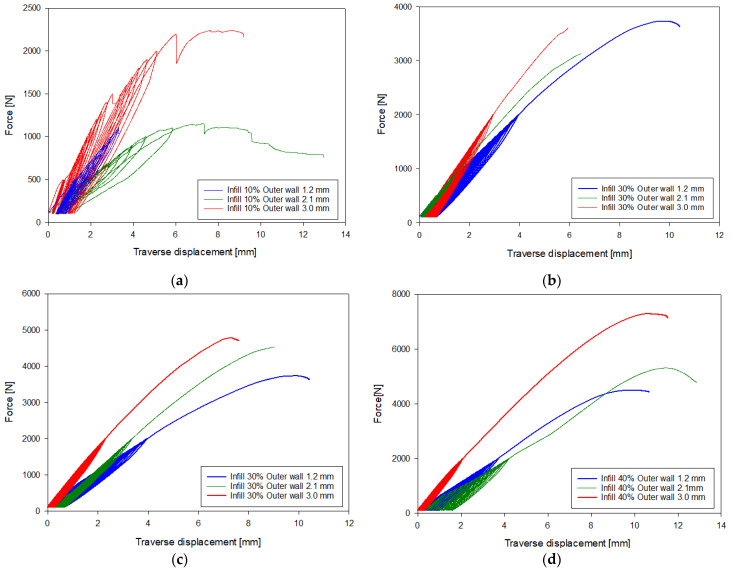
Force-displacement graph of the traverse with (**a**) 10% Infill, (**b**) 20% Infill, (**c**) 30% Infill, and (**d**) 40% Infill.

Figure 4 shows force–traverse displacement graphs obtained during the uniaxial compression process of the printed bones. Table 2 presents a comparison of all tested bones based on the relationship between maximum force and inner layer filling.

**Figure 5:**
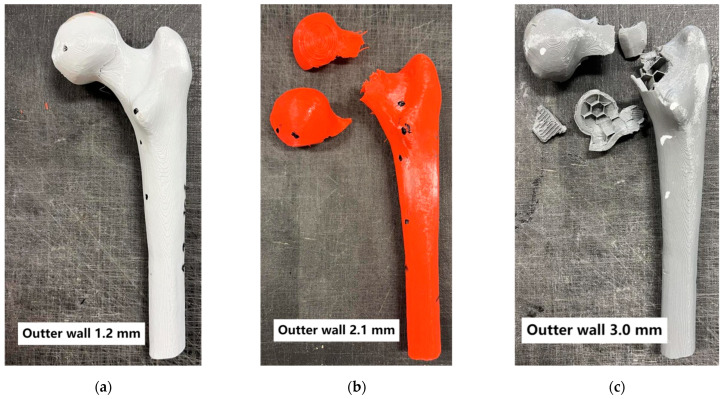
Bones with 10% internal layer filling.

**Figure 6:**
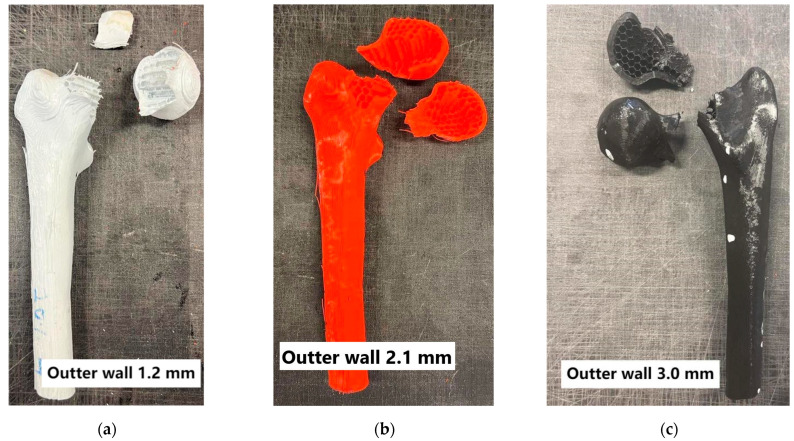
Bones with 20% internal layer filling.

**Figure 7:**
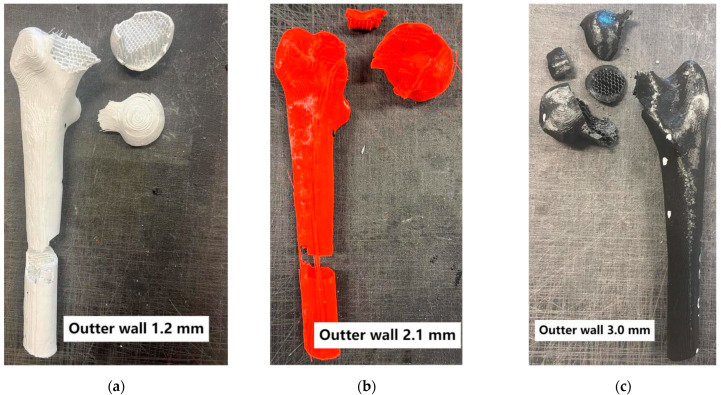
Bones with 30% internal layer filling.

**Figure 8:**
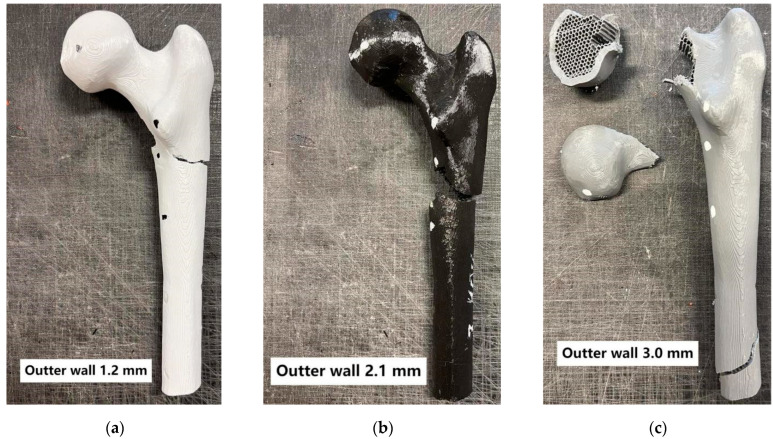
Bones with 40% internal layer filling.

Additionally, the fracture patterns of the femur were examined depending on changes in parameters such as the outer layer thickness, mimicking cortical bone, and the variation in the infill density of the inner layer, mimicking cancellous bone (Figure 5, Figure 6, Figure 7 and Figure 8).

### Analysis of 3D-Printed Bone

Analyzing the conducted laboratory experiments, it can be observed that both the outer layer thickness and the density of the inner layer have a significant impact on the overall load-bearing capacity of the analyzed bone model, as shown in Figure 5, Figure 6, Figure 7 and Figure 8 and Table 2. The ultimate compressive force was observed in the sample with an outer layer thickness of 3.0 mm and 40% internal infill, with 7291 N. The sample with the lowest compressive force was the one with 10% internal infill and outer layer thicknesses of 1.2 mm, with 1073 N, and 2.1 mm, with 1110 N. Another conclusion arising from the analysis of these attachments is the similarity in the ultimate force between different variants of the printed model. Examples of such variant pairs include an outer layer thickness of 1.2 mm and a 30% internal infill, and outer layer thickness of 3.0 mm and a 20% internal infill, an outer layer thickness of 1.2 mm and 40% internal infill, an outer layer thickness of 2.1 mm and 30% internal infill, an outer layer thickness of 1.2 mm and 20% infill, and an outer layer thickness of 3.0 mm and 10% infill. This suggests the possibility of achieving similar compressive strength under cyclic loading with different combinations of outer layer thickness and internal layer infill density. When comparing the ultimate force within a group of samples with the same outer layer thickness, it can be observed that each increase in the density of the internal layer infill results in a rise in the maximum force by approximately 1000 N. Based on Table 2, it can be concluded that both changes in the outer layer thickness and modifications in the infill thickness result in a linear change in the value of the destructive force.

A simplified stiffness analysis of the examined composite structure was also conducted. The stiffness of the system, denoted as k, was identified based on the slope of the Force–Displacement curve, in accordance with the methodology described in the literature [36,37,38]. This relationship was established for the first loading cycle within the force range of 100–500 N. The obtained stiffness values for the internal infill varied as follows: for 10% infill, the range was 308–677 N/mm; for 20% infill, 342–678 N/mm; for 30% infill, 580–902 N/mm; and for 40% infill, 586–985 N/mm. The lower values in each group correspond to an external layer thickness of 1.2 mm, while the upper values correspond to a thickness of 3.0 mm. A clear division in stiffness values can be observed. The first group includes the 10% and 20% infill levels, where both lower and upper values are similar. The second group comprises the 30% and 40% infill levels, which also yield comparable stiffness values within their range. This suggests that the external layer thickness has a significantly greater impact on the overall structural stiffness than the degree of infill within each group.

Failure in most of the tested variants occurred by fracturing the neck of the femoral head at its proximal end. Exceptions include variants with 40% internal infill and outer layer thicknesses of 1.2 mm and 2.1 mm, where destruction occurred by fracture below the lesser trochanter of the femur, and in the second case, by fracturing the femoral shaft. Analyzing the destruction pattern within a group with the same infill density, it can be observed that at 30% and 40% infill, the destruction of the sample affects not only the femoral head at its proximal end but also leads to a fracture in the femoral shaft, which was not observed in the cases with 10% and 20% infill. Referring to the previously mentioned variant pairs that exhibit similar compressive strength despite different combinations of parameters, shows that the destruction patterns are not always the same. The exception is the pair of samples with an outer layer thickness of 1.2 mm and 20% internal infill, and outer layer thickness of 3.0 mm and 10% internal infill, which were destroyed, similarly, by fracturing the neck of the femoral head at its proximal end. Analyzing the destruction pattern within a group with the same outer layer thickness, it can be observed that as the thickness increases, the head of the femur at its proximal end undergoes fragmentation into a greater number of pieces compared to the case with a smaller outer layer thickness at the same internal infill density. An example of this can be seen in the variant with 10% internal infill, where, in the case of an outer layer thickness of 1.2 mm, the head remains in one piece, with 2.1 mm it splits into two parts, and with an outer layer thickness of 3.0 mm, the head breaks into many smaller fragments. This may indicate an increased stiffness caused by the increased thickness of the cortical bone part. Although increasing both the outer layer thickness and the internal layer infill positively affects the stiffness of the model, as shown in Figure 4 and the maximum compressive force in Table 2, it is not possible to unambiguously assign a specific destruction pattern to a given combination. Different combinations of variables lead to different destruction patterns, which are difficult to predict with certainty. However, destruction patterns can be divided into two groups: A) Destruction of the femoral head or areas of the proximal femoral head. B) Destruction of the femoral head or areas of the proximal femoral head, along with a fracture in the femoral shaft region. Pattern 1 occurs with lower internal layer infill densities of 10–20%, while pattern 2 appears with higher internal layer infill densities of 30–40%.

Table 3 shows the distribution of the relationship between maximum force and the length of material used to print the samples. The length of this material is given, excluding the material needed for auxiliary supports created during the 3D printing process.

Analyzing the material consumption required to print the samples, the sample with an outer layer thickness of 3.0 mm and 40% infill required the most material, with 62.34 m, while the sample with an outer layer thickness of 1.2 mm and 10% infill consumed the least, with 36.63 m. Comparing these two factors, maximum force and material consumption, it can be clearly stated that the sample with an outer layer thickness of 1.2 mm and 10% infill is more economical than the sample with an outer layer thickness of 2.1 mm, providing similar compressive strength with lower material consumption.

## 4. Discussion

The aim of the present study was to evaluate how changes in outer layer thickness and variations in the density of the inner layer affect the overall strength of a 3D-printed human femur model. The obtained results for compressive strength and failure mechanisms were compared with commercially available artificial femoral bones and with human femoral bones. An additional advantage of this work is the assessment of 3D printing’s potential to customize the geometry of the printed model to meet the individual needs of potential patients.

### 4.1. Three-Dimensional-Printed Femur Bones by Other Authors vs. Three-Dimensional-Printed Bones

Comparing femur bones printed using 3D technology with those presented in the study by Nägl et al. [32], we can conclude that the maximum compressive force values obtained in our study fall within the general range of values reported by the aforementioned authors. The force values for the bones printed by Nägl et al. [32] ranged from 5500 N to 11,000 N, whereas in our study, the values ranged from 1100 N to 7300 N, depending on the printed variant. The failure pattern of the bones described by Nägl et al. is very similar to that observed in our study. In both cases, failure occurred due to the destruction of the proximal femoral head, fracture of the femoral neck, or failure of the shaft near the proximal femoral head. An ideal comparison of the failure pattern can be made between the SAW bone variant printed by Nägl et al. and the variant printed for this study with an outer thickness of 2.1 mm and 10% or 20% inner infill, as well as the variant with an outer thickness of 3.0 mm and 20% infill. In both cases, the femoral head fails in the neck region, and additionally, the neck itself is split into two parts. Another comparable failure pattern is observed between the printed SYN bone from the referenced study and the combination of 3.0 mm outer thickness with 10% infill and 3.0 mm outer thickness with 30% infill. In this case, the femoral head fails, but it is torn into multiple fragments of varying sizes.

### 4.2. Commercial Bones vs. 3D-Printed Bones

Comparing the maximum compressive force results obtained for commercial bones ORTHObones Premium Oberschenkelknochen (OB), ORTHObones Standard Oberschenkelknochen (OBS), SYNBONE^®^, SYN (right femur with distal canal opening 2230), and Sawbone (SAW, Femur, 4th Gen., Composite, 17 PCF Solid Foam Cancellous, Medium, SKU: 3403) subjected to uniaxial compression of the proximal femoral head, we observe comparable force ranges for selected combinations, following the study by Nägl et al. [32] and the results obtained in our study. The commercial bones described in the literature [33] achieved force values in the range of 1300 N–3400 N, which closely aligns with the results obtained for bones with 10% and 20% infill density of the inner layer in every outer layer thickness configuration. An exception is the SAW bone, which in the cited literature reached a maximum force value of 9100 N, whereas in our case, the highest value was 7300 N for an outer layer thickness of 3.0 mm with 40% inner infill density. The result obtained by the authors of [32] for the commercial SAW bone could potentially be achieved by increasing the density of the inner layer while maintaining the outer thickness at 3.0 mm. The failure pattern in commercial bones is also similar to that observed in our study. The bones failed due to shaft fracture, femoral neck fracture, or femoral head fracture.

### 4.3. Human vs. 3D-Printed Bones

Comparing the results obtained in this study with the ranges reported in the literature for human bones, it can be concluded that the obtained values fall within the range of human bone values. Ignoring the influence of donor gender on bone strength [35], the maximum compressive force values range on average from 3500 N [39] to 6600 N (with a broad range from 3780 to 12,396 N) [40]. Research results indicate significant variability in the mechanical properties of human bones, with values reaching up to 9196 ± 3177 N [41] and 8890 ± 3770 N [42]. Factors contributing to this variation may include gender, the degree of osteoporosis, or differences in testing setup. E. Dall’Ara et al. [43], when testing the femurs of three donors, obtained results of 13,620 N, 8568 N, and 4992 N, further confirming the possibility of obtaining results within a wide range.

Regarding the fracture patterns of human bones, the literature describes typical fractures of the proximal femur: subcapital, femoral neck, trochanteric, atypical fractures in the femoral head, or ambiguous fractures [43]. Dianna D. Cody et al. [41] identified three fracture patterns that correspond to some of the cases presented in this study: subcapital fractures, fractures extending into the mid-neck region, and oblique fractures. Iori G et al. [40] also indicated that the fracture location depends on the applied load, occurring in the subcapital and femoral neck regions.

The models of the femur with simplified geometry were analyzed because the thickness of the cortical bone of the femur varies depending on the region. Due to the limitations of the Zortrax M300 Plus printer, a uniform outer layer thickness is required around the entire contour of the model, and the “outer wall” layer thickness can be adjusted in the range from 0.3 mm to 3 mm, in increments of 0.3 mm. Additionally, the Zortrax M300 Plus printer only supports basic infill patterns, and the infill density can be adjusted from 0% to 100%, in increments of 10%. These studies were the first in this thematic area and serve as an introduction to more geometrically advanced models.

## 5. Conclusions

In the conducted study, an attempt was made to simulate various femur bone variants in terms of material characteristics. Three-dimensional printing technology offers the possibility of quick, cost-effective, and fully customizable manipulation of parameters that can replicate the actual tissue of the patient. The wide range of filaments and flexibility in modelling allows for an accurate representation of a patient’s actual condition. In this study, adjusting the outer layer thickness, which mimics cortical bone tissue, and modifying the inner layer density, which represents trabecular bone, led to the creation of various failure patterns that correlate with those observed in commercial and human bones. The obtained results demonstrate that, through the use of 3D printing technology, it is possible to achieve outcomes comparable to those observed in real bones, even when using a simplified bone model. The observed fracture mechanisms, which are characteristic of human femoral fractures, may be applied in orthopedic surgery for preoperative training purposes. The analyzed models can serve as training tools for current fracture stabilization techniques or as cost-effective and readily accessible alternatives for testing innovative fixation methods. As the scope of the research expands and more anatomically accurate models are implemented, the possibility of simulating osteoporotic conditions is also anticipated. Three-dimensional printing technology sets a new direction for patient-specific medicine, which could play a crucial role in orthopedics and surgery in the future. Personalized models, prepared within just a few hours, will undoubtedly assist both experienced and less experienced doctors in their preparation for surgeries and procedures. Printing a bone model with geometry and characteristics very close to the patient’s actual bone certainly creates enormous opportunities for physicians. Prior preparation of surgeons for a procedure or surgery could streamline the process and help prevent unwanted complications. Another application is in training young orthopedic surgeons, who often train using a limited set of available models. Here, we can generate the geometry not only of the femur but of any other bone in a fully controlled way, thus offering an unlimited range of complex case variants to help prepare young orthopedic doctors. The production of personalized orthoses and implants [5,6,7,8,11,44] also represents a potential development pathway for the application of 3D printing technology in medicine. Certainly, the current research serves as a foundation for further studies, which will be reflected in future articles on the application of 3D printing technology in medicine.

## Figures and Tables

**Table 1:** Selected mechanical properties of EcoLine PLA material.

Parameter	Value
Hardness in shore scale	81 D
Shrinkage	~0 [%]
Elongation at break	4.83 [%]
Strength at break	528.8 [N]
Resilience for V-Notch	0.29 J/cm^2^
Young Modulus	1471.93 [MPa]
Tensil at break	55.25 [MPa]
Print temp	190–230 °C
Heated bed	Not require
Nozzle	0.2–0.4 [mm]
Cooling	0–60 [%]
Flow	100 [%]

**Table 2:** Summary of printed bone variants.

Cortical Layer Thickness	Axial Strength of Bone [N]			
	10%	20%	30%	40%
1.2 mm	1073	2116	3727	4492
2.1 mm	1110	3124	4519	5298
3.0 mm	2240	3595	4779	7291

**Table 3:** Summary of the length of material used for each sample.

Cortical Layer Thickness	Length of the Used Material [m]			
	10%	20%	30%	40%
1.2 mm	36.63	41.05	46.74	53.03
2.1 mm	40.21	45.20	50.76	56.79
3.0 mm	47.05	51.85	56.95	62.34

## Data Availability

The original contributions presented in this study are included in the article. Further inquiries can be directed to the corresponding author(s).

## References

[1] Berman B. (2012). 3-D printing: The new industrial revolution. Bus. Horiz..

[2] Godoi F.C., Parakash S., Bhandari B.R. (2016). 3D printing technologies applied for food design: Status and prospects. J. Food Eng..

[3] Lim C.W.J., Le K.Q., Lu Q., Wong C.H. (2016). An overview of 3D printing in manufacturing, aerospace, and automotive industries. IEEE Potentials.

[4] Chen R.K., Jin Y., Wensman J., Shih A. (2016). Additive manufacturing of custom orthoses and prostheses—A review. Addit. Manuf..

[5] Dombroski C.E., Balsdon M.E., Froats A. (2014). The use of a low cost 3D scanning and printing tool in the manufacture of custom-made foot orthoses:a preliminary study. BMC Res. Notes.

[6] Telfer S., Pallari J., Munguia J., Dalgarno K., McGeough M., Woodburn J. (2012). Embracing additive manufacture: Implications for foot and ankle orthosis design. BMC Musculoskelet. Disord..

[7] Salles A.S., Gyi D.E. (2013). An evaluation of personalised insoles developed using additive manufacturing. J. Sports Sci..

[8] Ghai S.I., Sharma Y., Jain N., Satpathy M., Pillai A.K. (2018). Use of 3-D printing technologies in caraniomaxillofacial surgery: A review. Oral Maxillofac. Surg..

[9] Murphy S., Atala A. (2014). 3D bioprinting of tissues and organs. Nat. Biotechnol..

[10] Ventola C.L. (2014). Medical Applications for 3D Printing: Current and Projected Uses. Pharm. Ther..

[11] Murr L.E., Gaytan S.M., Medina F., Lopez H., Martinez E., Machado B.I., Hernandez D.H., Martinez L., Lopez M.I., Wicker R.B. (2010). Next-generation biomedical implants using additive manufacturing of complex, cellular and functional mesh arrays. Philos. Trans. A Math. Phys. Eng. Sci..

[12] Rengier F., Mehndiratta A., von Tengg-Kobligk H., Zechmann C.M., Unterhinninghofen R., Kauczor H.U., Giesel F.L. (2010). 3D printing based on imaging data: Review of medical applications. Int. J. Comput. Assist. Radiol. Surg..

[13] Wang X., Ao Q., Tian X., Fan J., Wei Y., Hou W., Tong H., Bai S. (2016). 3D Bioprinting Technologies for Hard Tissue and Organ Engineering. Materials.

[14] Ngo T.D., Kashani A., Imbalzano G., Nguyen K.T.Q., Hui D. (2018). Additive manufacturing (3D printing): A review of materials, methods, applications and challenges. Compos. Part B Eng..

[15] Choudhari C., Patil V. (2016). Product development and its comparative analysis by SLA, SLS and FDM rapid prototyping processes. IOP Conf. Ser. Mater. Sci. Eng..

[16] F42 Committee (2012). Standard Terminology for Additive Manufacturingtechnologies.

[17] Council N.R. (2004). Accelerating Technology Transition: Bridging the Valley of Death for Materials and Processes in Defense Systems.

[18] Wohlers T. (2017). Wohlers Report 2017: 3D Printing and Additive Manufacturing State of the Industry: Annual Worldwide Progress Report.

[19] Yu W., Sun X., Meng H., Sun B., Chen P., Liu X., Zhang K., Yang X., Peng J., Lu S. (2017). 3D-printed Porous Ceramic Scaffolds for Bone Tissue Engineering: A Review. Biomater. Sci..

[20] Khoshnevis B. (2004). Automated construction by contour crafting—Related robotics and information technologies. Autom. Constr..

[21] Zadpoor A.A., Malda J. (2017). Additive Manufacturing of Biomaterials, Tissues, and Organs. Ann. Biomed. Eng..

[22] Jia W., Gungor-Ozkerim P.S., Zhang Y.S., Yue K., Zhu K., Liu W., Pi Q., Byambaa B., Dokmeci M.R., Shin S.R. (2016). Direct 3D bioprinting of perfusable vascular constructs using a blend bioink. Biomaterials.

[23] Cui X., Breitenkamp K., Finn M.G., Lotz M., D’Lima D.D. (2012). Direct Human Cartilage Repair Using Three-Dimensional Bioprinting Technology. Tissue Eng. Part A.

[24] Keriquel V., Guillemot F., Arnault I., Guillotin B., Miraux S., Amédée J., Fricain J.-C., Catros S. (2010). In vivo bioprinting for computer- and robotic-assisted medical intervention: Preliminary study in mice. Biofabrication.

[25] Zopf D.A., Hollister S.J., Nelson M.E., Ohye R.G., Green G.E. (2013). Bioresorbable Airway Splint Created with a Three-Dimensional Printer. N. Engl. J. Med..

[26] Cubo N., Garcia M., del Canizo J.F., Velasco D., Jorcano J.L. (2016). 3D bioprinting of functional human skin: Production and in vivo analysis. Biofabrication.

[27] Keriquel V., Oliveira H., Remy M., Zaine S., Delmond S., Rousseau B., Rey S., Catros S., Amedee J., Guillemont F. (2017). In situ printing of mesenchymal stromal cells, by laserassisted bioprinting, for in vivo bone regeneration. Cartilage.

[28] Burkhard M., Fürnstahl P., Farshad M. (2019). Three-dimensionally printed vertebrae with different bone densities for surgical training. Eur. Spine J..

[29] Clifton W., Pichelmann M., Vlasak A., Damon A., ReFaey K., Nottmeier E. (2020). Investigation and Feasibility of Combined 3D Printed Thermoplastic Filament and Polymeric Foam to Simulate the Cortiocancellous Interface of Human Vertebrae. Sci. Rep..

[30] Clifton W., Damon A., Valero-Moreno F., Marenco-Hillembrand L., Nottmeier E., Tubbs R.S., Fox W.C., Pichelmann M. (2020). Investigation of the “Superior Facet Rule” Using 3D-Printed Thoracic Vertebrae with Simulated Corticocancellous Interface. World Neurosurg..

[31] Metzner F., Neupetsch C., Carabello A., Pietsch M., Wendler T., Drossel W.G. (2022). Biomechanical validation of additively manufactured artificial femoral bones. BMC Biomed. Eng..

[32] Nägl K., Reisinger A., Pahr D.H. (2022). The biomechanical behavior of 3D printed human femoral bones based on generic and patient-specific geometries. 3D Print Med..

[33] Kumar R.R., Rajesh D., Kumaran S., Ranjieth S., Ali M.I., Karthik K. (2022). Investigation on tensile characteristics of femur bone 3D model by using FDM. Mater. Today Proc..

[34] Reznikov N., Alsheghri A.A., Piché N., Gendron M., Desrosiers C., Morozova I., Sanchez Siles J.M., Gonzalez-Quevedo D., Tamimi I., Song J. (2020). Altered topological blueprint of trabecular bone associates with skeletal pathology in humans. Bone Rep..

[35] Bini F., Pica A., Marinozzi A., Marinozzi F. (2019). Prediction of Stress and Strain Patterns from Load Rearrangement in Human Osteoarthritic Femur Head: Finite Element Study with the Integration of Muscular Forces and Friction Contact. New Developments on Computational Methods and Imaging in Biomechanics and Biomedical Engineering.

[36] Chon C.-s., Yun H.-s., Kim H.S., Ko C. (2017). Elastic Modulus of Osteoporotic Mouse Femur Based on Femoral Head Compression Test. Appl. Bionics Biomech..

[37] Gardner M.P., Chong A.C., Pollock A.G., Wooley P.H. (2010). Mechanical evaluation of large-size fourth-generation composite femur and tibia models. Ann. Biomed. Eng..

[38] Heiner A.D., Brown T.D. (2001). Structural properties of a new design of composite replicate femurs and tibias. J. Biomech..

[39] Miura M., Nakamura J., Matsuura Y., Wako Y., Suzuki T., Hagiwara S., Orita S., Inage K., Kawarai Y., Sugano M. (2017). Prediction of fracture load and stiffness of the proximal femur by CT-based specimen specific finite element analysis: Cadaveric validation study. BMC Musculoskelet Disord..

[40] Iori G., Peralta L., Reisinger A., Heyer F., Wyers C., van den Bergh J., Pahr D., Raum K. (2020). Femur strength predictions by nonlinear homogenized voxel finite element models reflect the microarchitecture of the femoral neck. Med. Eng. Phys..

[41] Cody D.D., Gross G.J., Hou F.J., Spencer H.J., Goldstein S.A., Fyhrie D.P. (1999). Femoral strength is better predicted by finite element models than QCT and DXA. J. Biomech..

[42] Link T.M., Vieth V., Langenberg R., Meier N., Lotter A., Newitt D., Majumdar S. (2003). Structure analysis of high resolution magnetic resonance imaging of the proximal femur: In vitro correlation with biomechanical strength and BMD. Calcif. Tissue Int..

[43] Dall’Ara E., Luisier B., Schmidt R., Kainberger F., Zysset P., Pahr D. (2013). A nonlinear QCT-based finite element model validation study for the human femur tested in two configurations in vitro. Bone.

[44] Mirzaali M.J., Zadpoor A.A. (2024). Orthopedic meta-implants. APL Bioeng..

